# Voluntary blood donation knowledge, attitude, and practice among adult populations of Hosanna Town, South Ethiopia: a community-based cross-sectional study

**DOI:** 10.3389/fpubh.2023.1141544

**Published:** 2023-06-09

**Authors:** Abdulhakim Mussema, Solomon Gebre Bawore, Tewodros Abebaw, Wegayehu Tadese, Melsew Belayineh, Abel Yirga, Tofik Mohammed, Abdurezak Mohammed Seid

**Affiliations:** ^1^Department of Medical Laboratory Science, Wachemo University, Hosanna, Ethiopia; ^2^Department of Medical Laboratory Science, Nigist Eleni Mohammed Memorial, Comprehensive Specialized Hospital, Hosanna, Ethiopia; ^3^Department of Internal Medicine, Arba Minch University, Arba Minch, Ethiopia

**Keywords:** knowledge, attitude, practice, voluntary blood donation, Hosanna, Ethiopia

## Abstract

**Introduction:**

Even though blood donation has increased in the past decades, it remains a challenge worldwide. An adequate blood supply can only be assured through voluntary blood donation. There is inadequate information on the level of blood donation practice in the current study area. This study aimed to assess the knowledge, attitude, practice, and associated factors toward voluntary blood donation among Hosanna town adult populations.

**Methods:**

A cross-sectional study was conducted from 1 May 2022 to 30 June 2022, on a total of 422 adult populations of Hosanna town. A simple random sampling technique was used to select the study subjects. Data were collected by face-to-face interviews using a structured pre-tested questionnaire. The levels of knowledge, attitude, and practice of participants toward voluntary blood donation were measured using a set of questions. Data were analyzed using SPSS version 25. Chi-square and odds ratios were calculated, and the results were presented with words and tables.

**Results:**

In this study, a total of 422 participants enrolled with a response rate of 96.6%. Of the total respondents, 204 (48.3%), 209 (49.5%), and 123 (29.15%) study participants had good knowledge, favorable attitude, and experience of blood donation, respectively. Participants' sex being male and having favorable attitudes were found to have significant associations with blood donation practice. Furthermore, it was found that male participants were more than two and a half times more likely to donate blood than female participants (AOR: 2.53; 95% CI: 1.54, 4.15). Those who had favorable attitudes were found more than three and a half times more likely to donate blood than those having unfavorable attitudes (AOR: 3.54; 95% CI: 1.32, 9.46)

**Conclusion and recommendations:**

A large proportion of adult populations had poor knowledge, unfavorable attitudes, and low practice toward voluntary blood donation. Therefore, local and national blood banks and transfusion agencies should design strategies that can be implemented to improve the knowledge or attitude of the adult population and motivate the population to donate blood voluntarily.

## Introduction

Millions of lives are saved every year through blood transfusions, indispensable and life-saving support. It is crucial for those with severe anemia brought on by traumatic injuries, accidents, complications during pregnancy, major surgeries, transplants, congenital hemoglobin abnormalities, and immune-deficiency diseases (1–3). As the theme of World Blood Donor Day 2022, “Donating blood is an act of solidarity. Join the effort and save lives.” (4). The need for blood is increasing universally, but access to safe blood remains a challenge worldwide (1, 2, 4).

The percentage of total blood donations is a good indicator of a nation's general blood supply. The World Health Organization (WHO) reported in 2018 that over 118.5 million units of blood were donated annually, with industrialized nations accounting for nearly 40% of these donations. Fifty-four countries received more than 50% of their blood supply from family/replacement or paid donors, even though 79 countries receive over 90% of their blood supply from volunteer unpaid blood donors (5). In general, the WHO noted that 60 nations collected fewer than 10 donations for every 1,000 citizens. The WHO's African Region includes 34 of these nations. Although the amount of voluntary unpaid donations has increased over the last few decades, it is still a difficult global issue (1, 5). Recent study findings indicate that Ethiopia still has a low rate of voluntary blood donations (6–8). Hence, encouraging people to donate blood voluntarily could help to ensure that there is enough safe blood available at all healthcare facilities (1, 5).

Age, sex, education level, degree of understanding, and attitude are some of the variables that have been found to have an impact on the practice of volunteer blood donation in earlier studies (6, 9–12). The only way to ensure a sufficient supply of blood is to increase voluntary blood donation programs at the national and local levels and to manage donors well (1, 4). To the best of our knowledge, there is insufficient information on the frequency of voluntarily donating blood and related factors in the context of the current study. Therefore, the purpose of this research was to assess adult populations in Hosanna town's knowledge, attitudes, practices, and factors related to voluntary blood donation.

## Materials and methods

### Study design and setting

A community-based cross-sectional study was conducted in Hosanna town, Southern Ethiopia, 132 km south of Addis Ababa. Hosanna town has one blood bank center and one tertiary teaching hospital.

### Study population, sample size determination, and sampling technique

The town of Hosanna has six kebeles (the administrative unit in Ethiopia, below the town level). Three gots (the smallest administrative unit in Ethiopia) were randomly chosen from each of these kebeles, with two of them being chosen by basic random sampling techniques. A single population proportion formula [*n* = [(Zα/2)^2^^*^ P (1 – P)]/d^2^] was used to determine the sample size by using a prior study's finding of a 48.5% frequency of favorable attitude toward blood donation (6). The sample size was determined to be 437 using 95% CI, a 5% margin of error, and taking a 15% non-response rate into account. By dividing the total number of households in each chosen got by the proportional sample size that was obtained for each got, the sampling interval (K) value for each got was determined. These estimations were used to determine the proportional allocation of 242 people from Arada and 195 people from the Hetto kebele. Using a systematic selection procedure, households were chosen proportionately from the chosen gots. Ultimately, suitable household members who were at least 18 years old and volunteers were chosen as study participants using simple random sampling approaches at the household level. A subject was not included in the study if they were suffering from a serious illness (impairment or physical or mental conditions that make the individuals unable to participate in the study/respond adequately) or were unable to communicate during the data collection.

### Study variables

#### Independent variables

Socio-demographic variables such as sex, age, educational status, marital status, religion, and different variables can measure the knowledge, attitude, and practice of participants toward voluntary blood donation.

#### Dependent variables

The dependent variables are awareness, attitude, and practice of blood donation.

### Data collection

Data were obtained through face-to-face interviews with trained data collectors using a structured pre-tested questionnaire designed following an exhaustive review of related literature. The questionnaire was initially written in English, then translated into the Hadiyya language, and finally back to English. Socioeconomic and demographic characteristics, knowledge questions, attitude questions, and practice questions are all included in the questionnaire's four sections. The participants' level of knowledge on the requirements for blood donation, the risks of blood donation, the best sources of blood donors, and other topics were assessed using a variety of questions. Individuals who answered knowledge questions with a mean or higher score were classified as having a good knowledge level; otherwise, they had a bad knowledge level. The attitude was assessed using questions on general attitudes toward blood donation and future blood donation intentions. Individuals were classified as having a favorable attitude if their attitude scores were higher than the mean, in all other cases an unfavorable attitude. Blood donation was considered to be a practice for those who had at least one donation experience.

### Data management and analysis

The supervisors and data collectors received training prior to the study. Each questionnaire was visually reviewed, coded, filled out using Epi Data, and then sent for analysis. Word summaries and tables were used to present the results of the descriptive analysis. The correlation between the independent and dependent variables was analyzed using binary logistic regression. The final multivariable logistic regression model was employed to identify characteristics that were independently associated with the practice of blood donation. Any variables with a *p*-value in the bivariate analysis < 0.25 were included in the final multivariable logistic regression. The data were presented using an adjusted odd ratio (OR) and its 95% confidence range. A *p*-value of 0.05 was chosen as the cutoff for a significant association.

### Ethical considerations

This study was carried out after receiving approval from Wachemo University's Research Review Committee. The Hosanna town administrator's office was then contacted to request permission. Each participant in the study was informed of its aim, gave written consent, and was given the option to withdraw at any point if they felt uncomfortable with the questionnaire. Furthermore, participants' privacy and confidentiality were protected.

## Results

### Socio-demographic characteristics of participants

Of the sample size determined, 422 (96.6%) of them agreed to participate in the study. The ages of respondents ranged from 21 to 60 years, with mean ± standard deviations (SD) of (35.31 ± 12.61). The majority, 201 (47.63%), of participants were found between the age group of 26 and 35 years. Of the respondents, 220 (52.13%) of them were female participants. Approximately 144 (34.12%) of the participants had primary education followed by a diploma or above 132 (31.28%). Regarding marital status, 266 (63.03%) of the respondents were married, and 234 (55.45%) of the participants were followers of the Protestant religion (Table 1).

**Table 1:** Socio-demographic characteristics of participants in the study of voluntary blood donation in Hosanna Town, 2022 (n = 422).

**Variables**	**Categories**	**Frequency**	**Percent**
Age categories	18–25	175	41.47
	26–35	201	47.63
	36–45	38	9
	>45	8	1.89
Sex of respondents	Male	202	47.87
	Female	220	52.13
Marital status of respondents	Single	127	30.10
	Married	266	63.03
	Widowed and divorced	29	6.87
Religion of respondents	Orthodox	106	25.12
	Muslim	79	18.72
	Protestant	234	55.45
	Other	3	0.71
Educational status of respondents	No formal education	20	4.74
	Primary education	144	34.12
	Secondary education	126	29.86
	Diploma and above	132	31.28
Occupational status of respondents	Government employee	105	24.88
	Farmer	26	6.16
	Housewife	89	21.09
	Student	48	11.37
	Daily labor	43	9.72
	Merchant	111	26.30
Monthly income	≤ 1,500 birr	163	39.8
	>1,500 birr	259	61.4

### Knowledge of study participants toward voluntary blood donation

According to the current study, (51.7%) Cl: (46.8–56.5%) of adult populations had inadequate awareness about blood donation. Out of the 422 participants, 389 (92.18%) had information about blood donation. All the study participants knew good health as the criteria for blood donation. Only 138 (32.70%) participants knew their blood type. Furthermore, only 198 (46.9%) of the participants were aware that they may donate blood every 3 months. Nearly half, 204 (48.3%), of the study participants had good knowledge about blood donation (Table 2).

**Table 2:** Blood donation knowledge levels among the adult population in Hosanna Town, South Ethiopia, 2022.

**Variable**		**Response**	**Frequency**	**Percent**
Had you heard about blood donation before the study		Yes	389	92.18
		No	27	6.40
		Not sure	6	1.42
Minimum criteria for donating blood	Age	< 18	3	0.7
		18–65	304	72.0
		>65	115	27.3
	Weight	< 45 kg	86	20.4
		>45 kg	336	79.6
	Good health	No	0	0
		Yes	422	100
Do you know the most blood group type		Yes	193	45.73
		No	229	54.26
Do you know your own blood group type		Yes	138	32.70
		No	284	67.30
Interval of blood donation		Once every 3 months	198	46.9
		Other (once a week, monthe, 6 month, years)	224	53.1
Amount of volume needed when donating blood		< 250 ml	12	2.84
		250–500 ml	86	20.38
		>500 ml		
		I Don't know	324	76.78
Time needed for donation		< 20 min	37	8.77
		>20 min	37	8.77
		No idea	348	82.46
Level of Knowledge		Poor knowledge	218	51.7
		Good knowledge	204	48.3

### Attitude of study participants toward voluntary blood donation

Of the total study participants in the study, 213 (50.5%) Cl: (45.6–55.3%) of them had a poor attitude toward blood donation. A total of 264 (62.62%) participants thought it was a good idea to donate blood, while 16 (3.76%) participants thought it was a poor idea. The remaining 78 (18.48%) of them were neutral. A total of 410 people (97.16%) thought that voluntary blood donation was the best way to obtain blood. From the total, 74 (17.54%) of the participants believed that risk will happen after donation, and 240 (56.87%) of participants were not certain about risk occurrence after donation. Of the total respondents, 407 (96.45%) agreed on his/her donation will encourage others to donate. Nearly 359 (85.07%) had an interest to donate in the future. However, just 198 (46.9%) of them intended to provide blood freely; instead, 216 (51.2%) of them wanted to give blood as a substitute if necessary. Nearly half of the respondents, 209 (49.5%), had a positive attitude toward voluntary blood donation (Table 3).

**Table 3:** Blood donation attitude levels among adults in Hosanna Town, South Ethiopia, 2022.

**Variables**	**Response**	**Frequency**	**Percent**
What do you think about voluntary blood donation?	Good	264	62.56
	Not good	16	3.76
	Not good, not bad	78	18.48
	I don't know	64	15.16
Best blood donation type	Voluntary blood donation	410	97.16
	Replacement blood donation	7	1.66
	Paid blood donation	4	0.95
	Self-blood donation	1	0.24
Do you think any risk happen after blood donation	Yes	74	17.54
	Will not happen	108	25.59
	Not sure	240	56.87
Your donation will encourage others to donate	Agree	407	96.45
	Disagree	15	3.55
Is family blood donation best for the same family	Yes	375	88.86
	No	4	0.95
	Not sure	43	10.19
Willingness to donate blood in the future	Yes	359	85.07
	No	63	14.93
Type of donation intended	Replacement	216	51.2
	Voluntary	198	46.9
	Paid	8	1.9
Level of attitude	Unfavorable attitude	213	50.5
	Favorable attitude	209	49.5

### Practice of study participants toward voluntary blood donation

Only 123 (29.15%) (Cl: 24.9–33.7%) of the study participants had ever donated blood, and of those, 114 (92.7%) had done it voluntarily and 9 (7.3%) had done so in place of family members. Only 19 (4.50%) of the participants who had previously donated blood had done so twice in their lifetime. On the other hand, 299 (70.85%) of the study's participants had never previously given blood. The most frequent reasons for not donating blood were that no one asked, fear of pain and anemia, and a phobia of the sight of blood (Table 4).

**Table 4:** Blood donation practice among the adult population in Hosanna Town, South Ethiopia, 2022.

**Variables**	**Response**	**Frequency**	**Present**
Had you donate blood	Yes	123	29.15
	No	299	70.85
Type of blood donation practiced	Replacement	9	7.3
	Voluntary	114	92.7
Frequency of donation	Never donated	299	70.85
	Once	104	24.64
	Twice or more	19	4.50
Reasons for not donating blood	Did not think of it	29	9.7
	No one asked for	162	54.2
	Fear of pain	142	47.5
	Fear of anemia	142	47.5
	Fear of weight loss	132	44.1
	Fear of sight of blood	113	37.8
	No reasons	14	4.7

### Factors associated with the practice of voluntary blood donation at Hosanna Town

To find variables that are independently associated with the practice of blood donation, variables with a *p*-value of 0.25 in a bivariate analysis were chosen and incorporated into the multivariable logistic regression model. Being male and having a positive attitude were found to be independently associated with the practice of blood donation in the multivariable logistic regression analysis. Male participants in the study were more than twice as likely as female participants to donate blood (AOR: 2.53; 95% CI: 1.54, 4.15). Furthermore, it was revealed that the practice of donating blood was independently associated with having a positive attitude. When compared to individuals with negative attitudes, those with positive attitudes were more than three times as likely to volunteer to give blood (AOR: 3.54; 95% CI: 1.32, 9.46) (Table 5).

**Table 5:** Bivariate and multivariable analyses of factors associated with the practice of voluntary blood donation in Hosanna Town, South Ethiopia, 2022.

**Variables**		**Ever donated**		**COR (95% CI)**	**AOR (95% CI)**	***P-*value**
		**Yes**	**No**			
Age category	18–25	44	131	1.01 (0.19–5.18)	1.49 (0.26–8.50)	0.662
	26–35	74	127	1.75 (0.34–8.89)	2.21 (0.40–13.18)	0.346
	36–45	3	35	0.26 (0.35–1.88)	0.21 (0.26–1.65)	0.137
	>45	2	6	1		
Sex of Respondents	Female	39	163		1	
	Male	84	136	2.58 (1.67–4.02)	2.53 (1.54–4.15)	< 0.001
Marital status of Respondents	Single	29	98	1		
	Married	91	175	4.51 (1.33–15.29)		
	Divorced and widowed	3	26	2.56 (0.72–9.09)		
Educational Status	No formal education	3	17	1		
	Primary education	53	91	3.30 (0.92–11.79)		
	Secondary education	31	95	1.85 (0.51–6.73)		
	Diploma and above	36	96	2.12 (0.59–76.69)		
Monthly income	≤ 1,500 birr	77	182	1		
	>1,500 birr	46	117	0.92 (0.61–1.43)		
Level of Knowledge	Poor Knowledge	42	184	1		
	Good Knowledge	81	115	3.01 (1.99–4.79)	1.30 (0.49–3.41)	0.60
Level of Attitude	Unfavorable attitude	37	176	1		
	Favorable attitude	86	123	3.33 (2.12.5.21)	3.54 (1.32–9.46)	0.012

## Discussion

Currently, the scarcity of blood in transfusion facilities is a major public issue worldwide. Many patients lack access to the safe blood transfusions required for complicated medical and surgical procedures. Efficient volunteer blood donation is achieved by improving the adult populations' knowledge, attitude, and practice toward volunteer blood donation. We attempted to assess the adult population's knowledge, attitude, and practice about voluntary blood donation in the southern Ethiopian town of Hosanna. In this study, it was found that 48.3, 49.5, and 29.15% of the participants had good knowledge, a favorable attitude, and experience with blood donation, respectively.

The prevalence of inadequate blood donation knowledge among adult populations (51.7%) (Cl: 46.8–56.5%) was similar to other related community-based research carried out in Harar town (56.45%) (8), Adama town (52.9%) (6), and Mekelle town (51%) (13) in Ethiopia. The result, however, was higher than a study finding reported in Gondar town (43.2%) (14) and Debre Markos town (43.5%) (7) and lower than another study finding reported in Gonder town (64.6%) (15) and Jordan (71.4%) (11). This difference may have been caused by a variation in how knowledge level is measured, as well as a difference in the time period, study setting, and sample size.

Of the total participants in the study, 213 (50.5%) (Cl: 45.6–55.3%) had a negative attitude toward blood donation. This result is in line with the studies conducted in Birbir town and Adama town, where 54.8% (16) and 47.1% of the study participants, respectively, had a poor attitude toward blood donation (6), which was higher than the study conducted in Gonder town, where 18% of participants have a poor attitude toward voluntary blood donation (8) but lower than another study finding in Harar town, where 67.1% (14) had a poor attitude toward blood donation. The discrepancy may have been caused by a difference in how the attitude level was measured, a difference in culture, a difference in time, or a variation in the environment in which the study was done.

The results of this study also revealed that 299 (70.85%) (Cl 66.3–75.1%) of the participants had never given blood in their lives. This study finding is comparable to that of Bale Robe town (73%) (17), higher than that of Hong Kong 50.5% (18), and lower than that of Harar town 77.4% (14), Gonder town 81.6% (14), Birbir town 89.4% (16), Debre Markos town (83.9%) (7), and Adama town 83% (6). The WHO advises that all nations obtain their blood from consenting blood donors (19). Over the past 10 years, Ethiopia has seen a marked increase in voluntary blood donation (20). Despite the country's reasonable voluntary blood donation rate, there is still insufficient blood available to stop the morbidity and mortality of children and women in particular. The possible explanation for the difference in those study findings might be due to the difference in the time period of study and the cultural difference of the populations in which the study was conducted.

According to this study, men are more than 2.5 times more likely than women to donate blood (AOR: 2.53; 95% CI: 1.54, 4.15), and those with positive attitudes were 3.54 times more likely to volunteer to donate blood than people with negative attitudes (AOR: 3.54; 95% CI: 1.32, 9.46) (Table 5). This was similar to other research that was published elsewhere (6, 9–11, 16, 21). In addition, the majority of study participants had never donated blood previously because no one had requested it, they were afraid of pain, they were afraid of anemia, and they were afraid of seeing blood. The reasons for not donating blood are comparable to those found in other Ethiopian studies (6, 14). Government and other stakeholders must take considerable public health measures to raise awareness of the value and associated risks of blood donation to ensure a steady supply and availability of safe blood for transfusions.

### Limitations and strengths of the study

The common limitations of such types of studies are that responses might be influenced by socially desirable traits, interviewer, and recall bias. The results of this study cannot be generalized to other populations in Ethiopia, which is another study's drawback. Multicultural societies may be strongly influenced by local customs and the sociodemographic characteristics of their populations. Finally, because this study was conducted in the community, it may provide a clear image of how the population in the community feels about donating blood voluntarily.

## Conclusion and recommendations

This study showed that a significant number of adult populations had inadequate information, a poor attitude, and low practice of voluntary blood donation. The reasons given for not donating blood included a lack of knowledge, fear of pain and anemia, and fear of blood. Moreover, sex and attitude level were statistically significant variables that had an impact on blood donation practices in this study. To increase the understanding and attitude of the adult population and encourage voluntary blood donation, local, national, and transfusion agencies should develop strategies that should be executed.

## Data availability statement

The original contributions presented in the study are included in the article/supplementary material, further inquiries can be directed to the corresponding author.

## Ethics statement

Written informed consent was obtained from the individual(s) for the publication of any potentially identifiable images or data included in this article.

## Author contributions

The study was designed by AM, SB, and AS. It was collected by TA, WT, MB, and AY. The manuscript was written after the data analysis by AM and AS. The manuscript was carefully reviewed and edited by AM and AS and finally approved by SB and AS. All authors contributed to the article and approved the submitted version.

## References

[1] World Health Organization. Towards 100% Voluntary Blood Donation: A Global Framework for Action. Geneva: World Health Organization (2010). 26225399

[2] Dobson GP. Addressing the global burden of trauma in major surgery. Front Surg. (2015) 2:43. 10.3389/fsurg.2015.00043 26389122 PMC4558465

[3] Loua A Nikiema JB Sougou A Kasilo OJM. Transfusion in the WHO African region. Transf Clin Biol. (2019) 26:155–9. 10.1016/j.tracli.2019.06.191 31255509

[4] World Health Organization. Unpaid, voluntary blood donation saves countless lives. 13 June 2022, press release of WHO 2022. (2022).

[5] World Health Organization. Blood safety and availability - World Health Organization. 26 May 2022 News release Geneva (2022).

[6] Alemayehu G. Voluntary Blood Donation Knowledge, Attitudes, and Practices in Central Ethiopia. Int J General Med. (2020) 13:67–76. 10.2147/IJGM.S246138 32184648 PMC7061424

[7] Jemberu YA Esmael A Ahmed KY. Knowledge, attitude and practice towards blood donation and associated factors among adults in Debre Markos town, Northwest Ethiopia. BMC Hematol. (2016) 16:23. 10.1186/s12878-016-0062-8 27602227 PMC5011944

[8] Urgesa K Hassen N Seyoum A. Knowledge, attitude, and practice regarding voluntary blood donation among adult residents of Harar town, Eastern Ethiopia: a community-based study. J Blood Med. (2017) 8:13–20. 10.2147/JBM.S121460 28243159 PMC5317315

[9] Getie A Wondmieneh A Getahun M Gedefaw G Demis A. Knowledge of blood donation and associated factors in Ethiopia: A systematic review and meta-analysis. BMJ Open. (2021) 11:e044343. 10.1136/bmjopen-2020-044343 34226213 PMC8258569

[10] Feleke BE. Determinants of voluntary blood donation in the city of Bahir Dar: A case-control study. Asian J Transfus Sci. (2022) 16:56–60. 10.4103/ajts.AJTS_115_17 36199409 PMC9528563

[11] Abderrahman B Saleh MYN. Investigating knowledge and attitudes of blood donors and barriers concerning blood donation in Jordan. Procedia Soc Behav Sci. (2014) 116:2146–54. 10.1016/j.sbspro.2014.01.535

[12] Burzynski ES Nam SL Voir RL. Barriers and motivations to voluntary blood donation in sub-Saharan African settings_ a literature review. ISBT Sci Ser. (2016) 11:73–81. 10.1111/voxs.12271

[13] Mirutse G Fisseha G Abebe L Birhanu ZB Alemayehu M. Intention to donate blood among the eligible population in Mekelle city, northern ethiopia: using the theory of planned behavior. Am J Health Res. (2014) 2:158. 10.11648/j.ajhr.20140204.19

[14] Melku M Terefe B Asrie F Enawgaw B Melak T Tsegay YG. Knowledge, attitude, and practice of adult population towards blood donation in Gondar Town, Northwest Ethiopia: A community based cross-sectional study. J Blood Transf. (2016) 2016:7949862. 10.1155/2016/7949862 27516920 PMC4969535

[15] Enawgaw B Yalew A Shiferaw E. Blood donors' knowledge and attitude towards blood donation at North Gondar district blood bank, Northwest Ethiopia: a cross-sectional study. BMC Res Notes. (2019) 12:729. 10.1186/s13104-019-4776-0 31694710 PMC6836355

[16] Addisu AG Sultan H Deginet T Addishiwot Z Samuel T. Assessment of knowledge, attitude and practice of voluntary blood donation and associated factors among residents of Birbir Town. J Commun Med Health Educ. (2017) 7:504. 10.4172/2161-0711.1000504

[17] Mohammed A Yassin A Adem Aliyi A. Voluntary blood donation practice and its associated factors among civil servants in Bale Robe town, Southeast Ethiopia, 2021. SAGE Open Med. (2022) 10:205031212211020. 10.1177/20503121221102099 35646360 PMC9134437

[18] Suen L Siu JY Lee Y Chan E. Knowledge level and motivation of Hong Kong young adults towards blood donation: A cross-sectional survey. BMJ Open. (2020) 10:e031865. 10.1136/bmjopen-2019-031865 31959604 PMC7045270

[19] Asif N Hassan K. Voluntary blood donation. J Islamabad Med Dental College. (2015) 5:1–2.

[20] Federal democratic republic of Ethiopia ministry of health. In: *Special Bulletin 16th Annual Review Meeting.* (2014).

[21] Tadesse T Berhane T Abraha TH Gidey B Hagos E Grum T. Blood donation practice and associated factors among health professionals in Tigray regional state public hospitals, northern Ethiopia. BMC Res Notes. (2018) 11:677. 10.1186/s13104-018-3786-7 30249306 PMC6154916

